# Comment on Rapone et al. Consensus Statement on Full-Arch Implant Rehabilitations: Evidence-Based Recommendations from the Italian Consensus Conference. *J. Clin. Med.* 2026, *15*, 3695

**DOI:** 10.3390/jcm15218378

**Published:** 2026-10-28

**Authors:** Federico Tirone

**Affiliations:** Clinica Odontoiatrica Salzano Tirone, 12100 Cuneo, Italy; federico.tirone@gmail.com

I read with great interest the Consensus Statement by Rapone et al. [1]. The authors should be commended for the considerable effort involved in translating a heterogeneous body of evidence into structured and clinically applicable recommendations for full-arch implant rehabilitation. Such initiatives are particularly valuable in a field in which treatment protocols remain variable and many clinical decisions cannot be informed by randomized evidence alone.

The purpose of this Comment is limited to two operational recommendations that, in my view, may benefit from clarification regarding the relationship between the available evidence and the strength and specificity of the resulting clinical guidance: the recommendation against freehand implant placement and the quantitative criteria proposed for distal cantilever design. These observations are not intended to question the overall value of the Consensus, but to clarify the evidentiary basis of specific recommendations and the role of expert judgment in their formulation. This distinction is clinically relevant because strongly worded terms such as “standard of care” or “not recommended” may influence everyday clinical decisions.

First, Table 3 in the original publication identifies fully guided static computer-assisted implant surgery (CAIS) as the “standard of care for full-arch cases”, while stating that “Freehand placement is not recommended” [1]. I do not question the clinical value of CAIS, particularly when anatomical constraints, prosthetically demanding implant positioning, or immediate-loading workflows make accurate transfer of a virtual treatment plan especially valuable. Nor is there reason to dispute the consistent evidence that computer-assisted approaches improve positional accuracy compared with freehand placement.

However, a close examination of the evidence cited in Section 3.3.5, which provides the rationale for the CAIS guidance subsequently summarized in Table 3, raises the question of whether that evidence supports the categorical statement that freehand placement is not recommended. Vercruyssen et al. [2] provide a narrative review of computer-supported planning and guided surgery showing that guided surgery reduces deviation between planned and final implant position and suggesting its use for selected indications, including minimally invasive surgery, optimization of implant positioning, and immediate restoration; the review does not formulate a general recommendation against freehand placement. Cinquini et al. [3], cited together with Morton et al. [4] when introducing the three CAIS approaches, provide a narrative review specifically focused on the clinical performance of zirconia-based implant-supported fixed complete dental prostheses. The ITI prosthodontic consensus report by Morton et al. [4] addresses six questions concerning implant number, tilted versus axial implants, placement/loading protocols, zirconia implants, and zirconia versus metal–ceramic restorations; it was not designed to compare static, dynamic, robotic, and freehand implant placement. The systematic review by Lin and Eckert [5], to which Section 3.3.5 attributes the numerical accuracy estimates of 1.11 mm coronally, 1.40 mm apically, and 3.51° angularly, concerns intentionally tilted versus axially positioned implants. Those numerical estimates appear instead to correspond to the systematic review and meta-analysis by Khaohoen et al. [6], whose outcome variables were positional discrepancies between planned and placed implants. Khaohoen et al. provide evidence regarding accuracy within computer-assisted workflows, but do not establish that freehand placement is clinically inappropriate.

The distinction between evidence favoring one approach and evidence supporting a categorical recommendation against another therefore appears central. Tattan et al. [7], in a systematic review and meta-analysis restricted to randomized controlled trials, found static computer-aided implant placement to be significantly more accurate than freehand placement, while short-term implant survival was high and did not differ significantly among modalities in the available trials, although the certainty of evidence for this outcome was low. Conversely, Abdelhay et al. [8] reported a significantly lower pooled implant failure rate with guided compared to freehand placement (2.25% vs. 6.42%; RR 0.29, 95% CI 0.15–0.58). This finding supports a potential clinical advantage of guided placement and deserves consideration. However, the review was not restricted to full-arch rehabilitation and included heterogeneous study designs, surgical protocols, and follow-up periods, with one retrospective cohort alone accounting for 78.6% of the meta-analytic weight. The authors also identified differences in operator experience and the small number of high-quality comparative studies as limitations, calling for further standardized randomized trials. Importantly, the authors themselves concluded that both guided and freehand implant placement were associated with high implant survival rates.

More recently, Tomar et al. [9], in an umbrella review of 13 systematic reviews and meta-analyses, confirmed the greater positional accuracy of guided surgery while concluding that freehand placement remains a reliable treatment approach, particularly in experienced hands. This review was likewise not restricted to full-arch rehabilitation and therefore does not resolve the present question directly.

Accordingly, the question is not whether CAIS is more accurate—it demonstrably is—or whether it may provide clinical advantages in selected circumstances. The narrower question is whether the available evidence provides a sufficient basis for the categorical statement that freehand placement is not recommended across full-arch rehabilitation as a whole. Evidence favoring one surgical approach does not necessarily establish the clinical inappropriateness of another.

This distinction is particularly relevant when considered alongside the contemporaneous First Global Consensus for Clinical Guidelines for the Rehabilitation of the Edentulous Maxilla [10]. The guideline recommends prosthetically driven guided implant placement wherever feasible, while explicitly maintaining that freehand placement remains appropriate when guided workflows are not feasible or offer no added value. This formulation does not imply equivalence between the two approaches; rather, it acknowledges that the appropriateness of the surgical modality remains dependent on the clinical context.

A methodological clarification may therefore be useful. In Table 1 of the Italian Consensus, Statement 4—with 94% agreement, high certainty of evidence and a strong recommendation—addresses tilted versus axial implants, whereas the CAIS/freehand recommendation appears subsequently in Section 3.3.5 and Table 3. The manuscript does not report a separate Delphi agreement percentage or GRADE assessment specifically for the statement that freehand placement is not recommended. Clarification as to whether this operational recommendation underwent an independent evidence appraisal and consensus vote, or should instead be interpreted as expert-derived guidance extending Statement 4, would help readers understand its intended strength and scope; the latter would remain a legitimate form of consensus guidance, provided that this basis is made explicit.

Second, there is no question that cantilever length is an important biomechanical determinant and that unnecessary distal extension should be minimized. The concern relates not to the relevance of cantilever length itself, but to the formulation of universal linear thresholds independently of other structural variables that determine the mechanical behavior and fracture resistance of the prosthetic framework.

Section 3.3.7 states that distal cantilever length should not exceed 1.5 times the anterior–posterior (A-P) implant spread; it recommends avoiding extensions greater than 15 mm in the mandible and 10 mm in the maxilla regardless of the calculated A-P spread; and it classifies an A-P spread below 5 mm as an absolute contraindication to distal cantilever extension [1]. The Consensus states that the 1.5 × A-P spread threshold derives from the biomechanical analyses of Bevilacqua et al. [11].

A detailed examination of the cited study does not appear to provide direct validation of that threshold. Bevilacqua et al. performed a three-dimensional finite element analysis on a four-implant maxillary fixed prosthesis. Four configurations were tested while prosthesis length remained constant: distal implant angulations of 0°, 15°, 30°, and 45° were associated, respectively, with cantilever lengths of 13, 9, 5, and 0 mm. Distal implant length also changed between configurations, from 13 mm to 15 mm and 18 mm. Thus, implant inclination, cantilever length and distal implant length were modified concurrently rather than independently. A-P spread was neither defined nor independently manipulated as an experimental factor; different cantilever-to-A-P-spread ratios were not systematically tested, and the 1.5 ratio was not evaluated as a biomechanical or clinical threshold. The authors themselves emphasized that the numerical values should be interpreted as biomechanical indications within the limitations of the finite element model rather than as absolute stress values, and attributed stress reduction to both cantilever reduction and increased implant length.

This distinction is consistent with the review by Walter and Greenstein [12], which specifically examined the use of A-P spread to determine distal cantilever length and concluded that the relationship is not linear; that additional variables including beam mechanics, arch location, implant number and distribution, prosthetic material and framework design must be considered; and that recommendations deriving cantilever length from A-P spread have not been prospectively validated.

For zirconia frameworks, experimental evidence further illustrates why a linear dimension alone cannot characterize structural resistance. Chong et al. [13] demonstrated significant effects of both connector cross-sectional dimensions and cantilever loading distance on fracture force. Alshahrani et al. [14] similarly investigated different combinations of occlusocervical thickness and cantilever length and found both variables to have a significant effect on load to fracture. Clinical observational studies have also investigated framework geometry beyond cantilever length. Tirone et al. [15] evaluated 180 zirconia full-arch prostheses and showed that framework fracture was related not only to cantilever length but also to distal connector cross-sectional area and screw-access-opening length. These observational data should not be interpreted as establishing universally valid dimensional thresholds; their relevance here is that they demonstrate a clinically measurable multidimensional relationship between cantilever extension and the geometry of the resistant framework.

Clarification of the direct evidentiary basis for the 1.5 × A-P spread ratio, the fixed 15-mm mandibular and 10-mm maxillary limits, and particularly, the designation of A-P spread below 5 mm as an absolute contraindication would therefore be valuable. A similar methodological issue arises from Statement 5 in Table 1—with 91% agreement, high certainty of evidence and a strong recommendation—which addresses prosthetic material selection, while these quantitative cantilever criteria appear subsequently in Section 3.3.7 and Table 3 without separate GRADE assessments or Delphi agreement percentages. Clarifying whether these values represent evidence-derived thresholds or expert-derived pragmatic guidance, extending the broader statement, would allow clinicians to interpret them appropriately; if they are primarily consensus-based criteria, this should be stated explicitly.

Consensus statements necessarily integrate published evidence with expert judgment where direct comparative evidence is incomplete. This is legitimate and clinically useful. Precisely because such documents can substantially influence clinical practice, however, maintaining a transparent distinction between evidence-supported directional recommendations, recommendations supported primarily by surrogate outcomes, and expert-derived operational thresholds is essential.

Accordingly, two clarifications would strengthen the Consensus: first, establishing whether the recommendation against freehand placement should be understood as an evidence-derived conclusion or as expert-consensus guidance, and second, identifying whether the proposed cantilever limits and A-P spread thresholds are empirically validated thresholds or expert-derived pragmatic criteria.

I again congratulate Rapone et al. for undertaking an ambitious and clinically relevant consensus process. Addressing these points would further strengthen the methodological transparency and clinical applicability of the Consensus.
